# EZH2 inhibition in multiple myeloma downregulates myeloma associated oncogenes and upregulates microRNAs with potential tumor suppressor functions

**DOI:** 10.18632/oncotarget.14378

**Published:** 2016-12-30

**Authors:** Mohammad Alzrigat, Alba Atienza Párraga, Prasoon Agarwal, Hadil Zureigat, Anders Österborg, Hareth Nahi, Anqi Ma, Jian Jin, Kenneth Nilsson, Fredrik Öberg, Antonia Kalushkova, Helena Jernberg-Wiklund

**Affiliations:** ^1^ Science for Life Laboratory, Department of Immunology, Genetics and Pathology, Rudbeck Laboratory, Uppsala University, Uppsala, Sweden; ^2^ Department of Laboratory Medicine, Division of Clinical Immunology, Karolinska University Hospital, Huddinge, Stockholm, Sweden; ^3^ Department of Medicine, Faculty of Medicine, University of Jordan, Amman, Jordan; ^4^ Department of Oncology-Pathology, Karolinska University Hospital, Solna, Stockholm, Sweden; ^5^ Department of Medicine, Unit of Hematology, Karolinska University Hospital, Huddinge, Stockholm, Sweden; ^6^ Department of Pharmacological Sciences and Oncological Sciences, Icahn School of Medicine at Mount Sinai, New York, NY, USA

**Keywords:** multiple myeloma, EZH2, H3K27me3, microRNA, UNC1999

## Abstract

Multiple Myeloma (MM) is a plasma cell tumor localized to the bone marrow (BM). Despite the fact that current treatment strategies have improved patients' median survival time, MM remains incurable. Epigenetic aberrations are emerging as important players in tumorigenesis making them attractive targets for therapy in cancer including MM. Recently, we suggested the polycomb repressive complex 2 (PRC2) as a common denominator of gene silencing in MM and presented the PRC2 enzymatic subunit enhancer of zeste homolog 2 (EZH2) as a potential therapeutic target in MM. Here we further dissect the anti-myeloma mechanisms mediated by EZH2 inhibition and show that pharmacological inhibition of EZH2 reduces the expression of MM-associated oncogenes; IRF-4, XBP-1, PRDM1/BLIMP-1 and c-MYC. We show that EZH2 inhibition reactivates the expression of microRNAs with tumor suppressor functions predicted to target MM-associated oncogenes; primarily miR-125a-3p and miR-320c. ChIP analysis reveals that miR-125a-3p and miR-320c are targets of EZH2 and H3K27me3 in MM cell lines and primary cells. Our results further highlight that polycomb-mediated silencing in MM includes microRNAs with tumor suppressor activity. This novel role strengthens the oncogenic features of EZH2 and its potential as a therapeutic target in MM.

## INTRODUCTION

Multiple myeloma (MM) is a malignancy of antibody secreting monoclonal plasmablasts/plasma cells (PCs), which accumulate within the bone marrow (BM) [1, 2]. MM exhibits high clonal heterogeneity, which has limited the clinical benefits of current treatment strategies [3, 4], and therefore MM remains a fatal disease. Genetic and epigenetic abnormalities as well as the BM microenvironment have been proposed as important factors in MM pathogenesis, response to treatment and relapse, and therefore, represent targets for improved therapy [5, 6].

Epigenetic mechanisms regulating gene expression are important in fundamental biological processes such as pluripotency, cellular differentiation and reprogramming [7, 8]. Deregulation of epigenetic mechanisms is also a hallmark of cancer and has been implicated in tumor initiation, progression, metastasis and response to treatment [9–11]. Thus, epigenetic modifiers should represent potential targets for therapy in cancer. The histone methyltransferase enhancer of zeste homolog 2 (EZH2) is the catalytic subunit of the polycomb repressive complex 2 (PRC2). It mediates epigenetic silencing of target genes via tri-methylation of histone H3 lysine residue 27 (H3K27me3) at regulatory loci and gene bodies [12–14]. Many PRC2 target genes have been categorized as related to cell cycle check-points and differentiation [15, 16], suggesting that EZH2 plays important roles in promoting cell proliferation and transformation.

EZH2 has, by us and others, been shown to be overexpressed in malignant PCs as compared with normal bone marrow PCs [17, 18]. While EZH2 controls the formation of H3K27me3, genetic inactivation of the H3K27 lysine (K)-specific demethylase 6A (KDM6A, UTX) is frequent in MM [19, 20]. In addition, EZH2 and H3K27me3 mark have been shown to support the oncogenic function of MMSET and its associated H3K36me2 mark in a defined subset of MM patients harboring the t(4;14) translocation [21, 22]. Recently, we could show that genes commonly silenced in MM, as compared with normal plasma cells, are PRC2 targets and that the histone methylation of these targets correlated with gene repression in advanced MM stages and in patients with poor survival [18, 23]. Moreover, we and others demonstrated that pharmacological inhibition of EZH2 using small specific chemical inhibitors is a potentially interesting therapeutic strategy in MM [23, 24]. Collectively, these data suggest an important role for EZH2 and H3K27me3 in MM pathogenesis and highlight EZH2 as a promising therapeutic target.

In this paper, we further explore the anti-proliferative effects of EZH2 inhibition in MM by using the selective inhibitor UNC1999. We now show that EZH2 inhibition by UNC1999 reduced MM cell viability and the expression of MM-associated oncogenes; IRF-4, XBP-1, PRDM1 (hereafter BLIMP-1) and c-MYC in MM cell lines. We here demonstrate that the observed downregulation of MM-associated oncogenes upon EZH2 inhibition correlates with upregulation of miRNA with tumor suppressor function; mainly miR-125a-3p, miR-320c. These miRNAs are predicted to target IRF-4, XBP-1 and BLIMP-1. Furthermore, we could validate that miR-125a and miR-320c are polycomb targets in MM cell lines and plasma cells derived from newly diagnosed MM patients. This study presents a novel mechanism of EZH2 action in MM pathogenesis by regulating the expression of miRNA genes with tumor suppressor functions and further supports the notion of EZH2 as potential therapeutic target in MM also indirectly affecting the expression of MM-associated oncogenes.

## RESULTS

### EZH2 inhibition downregulates MM-related oncogenes

**Figure 1:**
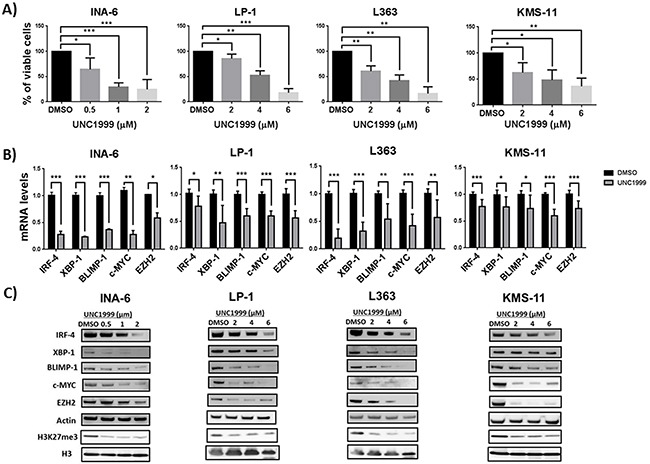
EZH2 inhibition reduces the viability of MM cells and downregulates the expression of MM-associated oncogenes **A.** EZH2 inhibitor UNC1999 reduces the viability of MM cells lines in a dose dependent manner. MM cell lines were treated with a range of UNC1999 concentrations and cell viability was assessed 5 days post-treatment using the AlamarBlue assay. **B.** EZH2 inhibition reduces the mRNA levels of IRF-4, XBP-1, BLIMP-1, c-MYC and EZH2 in MM cell lines upon treatment with UNC1999 for 5 days. mRNA levels were assessed after treatment with UNC1999 at concentration of 1 μM for INA-6 and 4 μM for the rest of the cell lines. Actin was used as housekeeping gene. C. EZH2 inhibition reduces the protein levels of IRF-4, XBP-1, BLIMP-1, c-MYC, EZH2 and global H3K27me3 levels in MM cell lines. Actin and total histone H3 were used as loading controls. The blots are representative of three independent biological experiments. Error bars represent standard deviation of three independent biological experiments. DMSO was used as control treatment. P-values were calculated using student t-test GraphPad prism, P-value: *≤ 0.05; **≤0.01; ***≤0.001.

Recently, we provided evidence that pharmacological EZH2 inhibition using UNC1999 exerts anti-MM effects by reducing the levels of H3K27me3 and de-repression of bivalent genes involved in tumor suppressor functions such as apoptosis and cellular differentiation [23]. In this study our aim was to extend our knowledge on the anti-MM effects mediated by EZH2 inhibition by focusing on the downregulated genes and to provide molecular mechanisms underlying this observation. To this end, we performed gene expression array on the MM INA-6 cell line treated with 1 μM of UNC1999 for 5 days. Using DMSO as control treatment, we could identify 530 differentially regulated mRNAs (P-value ≤ 0.02 and fold change of 1.5 cut off) among which 186 were upregulated and 344 were downregulated (Supplementary Table 1). The downregulated genes in this study were significantly enriched among the MM-unique H3K4me3 targets representing actively transcribed genes previously defined by us using ChIP-Seq [23] (Supplementary Figure 1A). Interestingly, we found that UNC1999 treatment resulted in a significant downregulation in the expression of MM-associated oncogenes; IRF-4, XBP-1, BLIMP-1 and c-MYC (Supplementary Figure 1B). QPCR and Western blot analysis confirmed that EZH2 inhibition using UNC1999 downregulated IRF-4, XBP-1, BLIMP-1 and c-MYC at the mRNA (Figure 1B) and protein (Figure 1C) levels in all MM cell lines tested in this study. We further showed that UNC1999 downregulated EZH2 at the mRNA and protein levels (Figure 1B and 1C), while EZH1 seemed to be less affected (Supplementary Figure 2A). Notably, kinetics of UNC1999 response in the most sensitive INA-6 cell line revealed that UNC1999 did not reduce MM-associated oncoprotein levels at earlier time points, 24 and 72 hours (Supplementary Figure 2B). Similar to our previous findings at 72 hours post-treatment with UNC1999 [23], we could show that UNC1999 inhibition of EZH2 reduced MM cell viability (Figure 1A) and global levels of the H3K27me3 mark (Figure 1C) at 120 hours post-treatment. We further confirmed that EZH2 inhibition resulted in upregulation of genes related to apoptosis such as ID2, ID3 and differentiation i.e. SOX2 (Supplementary Figure 1C) and downregulation of genes involved in metabolism and cell signaling (Supplementary Figure 1D). Induction of apoptosis over time was confirmed by a significant increase in activation of caspase-3 and -7 in two MM cell lines; INA-6 and KMS-11 (Supplementary Figure 2C). Importantly, caspase-3 and -7 activation was stronger in INA-6 than KMS-11 (Supplementary Figure 2C), which is also reflected by the percentage of viable cells where INA-6 was found to be more sensitive than KMS-11 (Figure 1A).

### EZH2 inhibition changes the miRNA expression profile in MM

EZH2 is the catalytic subunit of PRC2, a transcriptional repressive complex that mediates gene silencing via deposition of H3K27me3 mark [12–14]. Therefore, we asked how the inhibition of a transcriptional repressor reduces the expression of active genes such as IRF-4, XBP-1, BLIMP-1 and c-MYC. One plausible explanation was that EZH2, via H3K27me3, represses non-coding regulatory target genes i.e. miRNAs, which post-transcriptionally negatively modulate the expression of active genes. To investigate this hypothesis, we analyzed mature microRNA expression in the MM INA-6 cell line treated with 1 μM of UNC1999 for 5 days. Using DMSO treatment as control, we detected 206 miRNAs to be significantly (P-value ≤ 0.05 and fold change of 1.2 cut off) and differentially regulated by UNC1999; of which 118 were upregulated and 88 were downregulated (Supplementary Table 2). We found that EZH2 inhibition resulted in the downregulation of well-described oncomiRNAs in MM such as miRNAs belonging to the miR-17-92 cluster, miR-106b-25 cluster, and Let-7 family members (Table 1). In addition, EZH2 inhibition also led to upregulation of microRNAs reported to be underexpressed in MM as compared with normal plasma cells and with potential tumor suppressor functions. Of particular importance, miRNAs previously suggested to be epigenetically repressed via DNA methylation in MM were here shown to be upregulated upon EZH2 inhibition (Table 1).

**Table 1:** UNC1999 differentially regulated microRNAs that are relevant to MM based on previous observations

miRNA	Regulation	Comments	Refs
miRs-17-3P and 5p, miR-18a-5p, miR-18b-5P, miR-19a-3p, miR-19b-3p and 92a-3p	Down	OncomiRNAs; belongs to the miR-17-92 cluster; positively regulated by c-MYC and overexpressed in MM. members of the miR-17-92 cluster target SOCS1 and BIM.	[46, 47, 49]
Let-7c-5p, Let-7f-5p, Let-7g-5p and Let-7i-5p	Down	Members of the Let-7 oncomiRNA family; overexpressed in MM; promote angiogenesis	[49]
miR-106b and miR-25-3p	Down	OncomiRNA; belongs to the miR-106b-25 cluster; overexpressed in MM; controls P53 activity	[46, 48, 49]
miRs-125b	Down	OncomiRNA, targets P53.	[51]
miR-20a-5p and miR-148a	Down	Over expressed in MM compared with normal PCs with a prognostic value	[66]
miR-125a-3p, miR-198	Up	Downregulated in MM cell lines and patient samples; upregulated in MM cell lines upon treatment with 5´-aza	[46, 54–56]
miR-601, miR-765, miR-877-5p	Up	DNA hypermethylated in MM patients samples, upregulated in MM cell lines upon treatment with 5-aza-CdR	[44]
miR-1290, miR-223-3p, miR-320c, miR-630	Up	Upregulated in MM cell lines upon treatment with 5-aza-CdR	[44]

### MiR-125a-3p and miR-320c are predicted to target IRF-4, XBP-1 and BLIMP-1 transcripts

**Figure 2:**
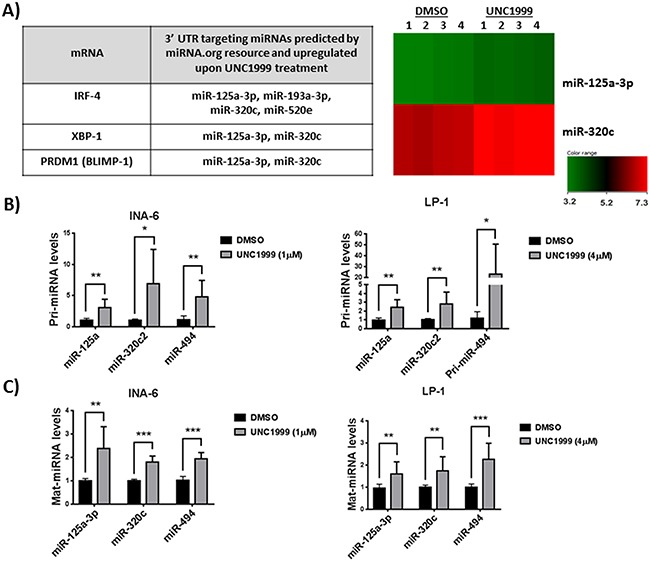
EZH2 inhibition induces the expression of miR-125a and miR-320c that are predicted to target IRF-4, XBP-1 and BLIMP-1 **A.** miRNAs predicted by miRNA.org resource to target IRF-4, XBP-1 and BLIMP-1 and to be upregulated by EZH2 inhibition in the MM INA-6 cell line. Heatmap analysis shows changes in expression of miR-125a and miR-320c in four independent biological experiments. INA-6 cell line was treated with 1μM of UNC1999 for 5 days. QRT-PCR validation of primary miRNA **B.** and mature miRNA **C.** in two MM cell lines, INA-6 treated with 1 μM of UNC1999 and LP-1 treated with 4 μM of UNC1999 for 5 days. Upregulation of miR-494 that is known to target c-MYC was detected by qRT-PCR but not in mature miRNA array. RNU6B was used as housekeeping miRNA. DMSO was used as control treatment. Error bars represent standard deviation of three independent biological experiments. P-values were calculated using student t-test GraphPad prism, P-value: *≤ 0.05; **≤0.01; ***≤0.001.

Having demonstrated that EZH2 inhibition reduces the expression of oncogenes either previously involved in genetic aberrations or strongly associated to growth in MM; IRF-4, XBP-1, BLIMP-1 and c-MYC, we used the microRNA.org resource [25] to identify which of the miRNAs predicted to target these genes were regulated by UNC1999. Focusing on the upregulated miRNAs, we identified two miRNAs: miR-125a-3p and miR-320c to be common regulators of IRF-4, XBP-1 and BLIMP-1 transcripts (Figure 2A). This analysis did not, however, find miRNAs predicted to target c-MYC to be regulated by UNC1999. Using qPCR, we confirmed the upregulation of miR-125a-3p and miR-320c at the primary (Figure 2B) and mature (Figure 2C) levels in the MM INA-6 and LP-1 cell lines. For the in depth analysis we included additional miRNAs previously shown to target c-MYC i.e. miR-126 and miR-494. By qPCR we show that EZH2 inhibition using UNC1999 resulted in the upregulation of miR-494 (Figure 2B and 2C), but not miR-126 (data not shown) in the MM INA-6 and LP-1 cell lines. Kinetics of primary microRNA expression in the INA-6 cell line revealed that UNC1999 induced the expression of primary microRNAs at 72 hours post-treatment, which peaked at 120 hours post-treatment with UNC1999 (Supplementary Figure 3).

### MiR-125a and miR-320c are targets of EZH2 and H3K27me3 in MM cell lines and primary cells

**Figure 3:**
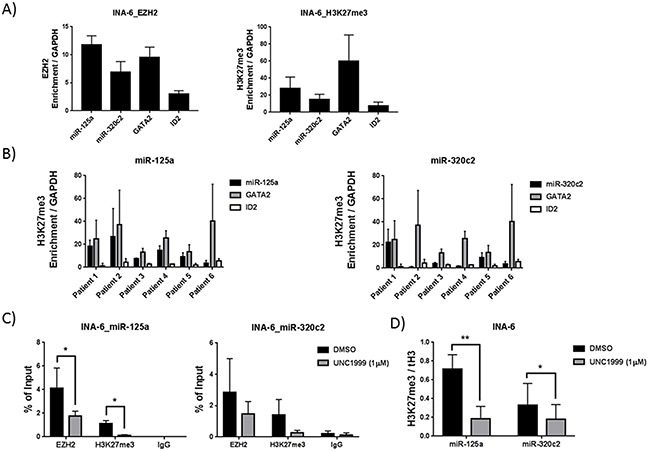
MiR-125a and miR-320c are polycomb targets in MM cell lines and primary cells as determined by ChIP-qPCR **A.** EZH2 and H3K27me3 enrichment at miR-125a and miR-320c gene bodies in the MM INA-6 cell line. **B.** H3K27me3 enrichment at the miR-125a and miR-320c gene bodies in CD138+ myeloma cells purified from newly diagnosed patients. GATA2 and ID2 promoter regions were used as positive control for enrichments, while GAPDH promoter region was used as negative control. Enrichment was represented relative to the negative control, GAPDH. **C.** EZH2 inhibition using UNC1999 reduces the occupancy of EZH2 and H3K27me3 mark at miR-125a and miR-320c gene bodies. **D.** Reduced levels of H3K27me3 mark at miR-125a and miR-320c upon UNC1999 treatment was independent of total histone H3 levels. INA-6 cell line was treated with 1 μM of UNC1999 for 5 days. DMSO was used as control treatment. Error bars represent standard deviation of three independent biological experiments for cell lines and two technical qPCR runs for patient samples. P-values were calculated using student t-test GraphPad prism, P-value: *≤ 0.05; **≤0.01.

To investigate whether miR-125a and miR-320c are direct polycomb targets in MM we performed chromatin immunoprecipitation followed by qPCR (ChIP-qPCR) to study EZH2 and H3K27me3 enrichment at the miRNA coding regions. Figures 3A and Supplementary Figure 4A show that both miR-125a and miR-320 genes are enriched for EZH2 and H3K27me3 in the MM INA-6 and LP-1 cell lines. We could also show that miR-125a and miR-320c are targets of H3K27me3 in purified CD138^+^ malignant plasma cells from newly diagnosed patients (Figure 3B). The enrichment of EZH2 and H3K27me3 mark at the miRNA regions was similar to that detected at the promoter regions of the control genes GATA2 or ID2, previously defined as PRC2 targets (Figures 3A, 3B and Supplementary Figure 4A). Upon treatment with the EZH2 inhibitor UNC1999 in MM cell lines, the enrichment of EZH2 and H3K27me3 mark at the analyzed regions were reduced (Figure 3C and Supplementary Figure 4B). Importantly, reduced levels of H3K27me3 mark upon EZH2 inhibition were not due to reduced total histone H3 levels (Figure 3D and Supplementary Figure 4C).

## DISCUSSION

Multiple Myeloma (MM) is a hematological malignancy characterized by the accumulation of malignant antibody producing plasmablasts/plasma cells in the bone marrow (BM) [1, 2]. Disease associated clonal and inter-patient heterogeneity has hampered identification of a common underlying mechanism for disease establishment and slowed the development of novel targeted therapies [3, 4, 26, 27]. We recently identified a common H3K27me3-marked chromatin profile mediated by EZH2 among MM patients that correlates to gene silencing in advanced stages of the disease and to poor clinical outcome [18, 23]. Furthermore, we and others have demonstrated that EZH2 provides a potential therapeutic target in MM by using highly selective inhibitors of EZH2 *in vitro* [23, 24] and *in vivo* [24]. Here we further investigated the anti-myeloma effects mediated by pharmacological inhibition of EZH2 by focusing on downregulated genes in MM and the molecular mechanisms underlying this observation.

In the present study, we performed gene expression array in the MM INA-6 cell line treated with UNC1999 for 5 days. In agreement with our previous gene expression profiling upon 72 hours inhibition of EZH2 [23], we found that EZH2 inhibition led to reactivation of genes involved in apoptosis and cell differentiation and downregulated genes related to cell signaling and metabolism. This further solidifies our proposed function of EZH2 in MM in repressing tumor suppressor genes involved in apoptosis and cell differentiation. Notably, long-term inhibition of EZH2 also significantly reduced the expression of non-PRC2 target genes. These genes were over-represented among actively transcribed genes harboring the H3K4me3 mark in MM patients as previously defined by us using RNA- and ChIP-Seq [23]. Interestingly, we found IRF-4, XBP-1, BLIMP-1 and c-MYC to be downregulated upon EZH2 inhibition. IRF-4, XBP-1 and BLIMP-1 are essential transcription factors promoting normal B-cell development by inducing germinal center exit and plasma cell differentiation [28–30]. In addition, these transcription factors have also been suggested to have a major impact on MM pathogenesis. Shaffer et al. demonstrated that MM cells are addicted to IRF-4 by showing an absolute requirement of IRF-4 for MM growth irrespective of the transforming genetic event [31]. The importance of XBP-1 and BLIMP-1 in MM pathogenesis has been demonstrated by their frequent upregulation in MM and as drivers of MM pathogenesis in murine models [32–34]. Deregulation of c-MYC expression is important for MM cells survival [35, 36] and has been implicated as one of the important events in disease progression from the pre-malignant MGUS to MM in both human [37] and in murine MM models [38]. Thus, our results imply EZH2 inhibition as a novel strategy for anti-MM therapy due to downregulation of MM-associated oncogenes.

We investigated the possibility that EZH2 regulates the transcription of non-protein coding PRC2 targets i.e. miRNAs as a potential underlying mechanism for the downregulation of IRF-4, XBP-1, BLIMP-1 and c-MYC upon EZH2 inhibition. In this study, the analysis of global expression profiling of mature microRNAs revealed 206 miRNAs to be differentially regulated by EZH2 inhibition of which 118 were upregulated and 88 were downregulated. Among these miRNA were candidates predicted to negatively regulate the above mentioned MM-associated oncogenes at the post-transcriptional level. MiRNAs are small endogenous non-coding single stranded RNAs of about 22 nucleotides that negatively regulate gene expression post-transcriptionally [39]. Aberrant miRNA expression and/or function in MM have been attributed to genetic lesions e.g. chromosomal translocations and copy number variations [40–43] as well as to deregulation in epigenetic mechanisms e.g. DNA methylation [44, 45]. So far, less is known regarding the role of polycomb and H3K27me3 in regulating miRNA expression.

Among the miRNAs reduced by EZH2 inhibition were miRNAs found to belong to the miR-17-92 cluster, miR-106b-25 cluster and Let-7 family members, previously reported to function as oncogenes in MM [46–50]. MiR-17-92 cluster has been suggested to function as oncomiRNA in MM, as members of this cluster were shown to target tumor suppressor genes such as the pro-apoptotic BIM [46, 47]. In a similar manner, miR-106b-25 cluster [46, 48] and miR-125b [51] have been shown to possess oncogenic properties through modulating the activity of the tumor suppressor gene P53. In collaboration with miR-17-92 cluster, the Let-7 family members have been denoted a role in enhancing MM angiogenesis [50]. Interestingly, miR-17-92 and miR-106b-25 clusters expression has also been suggested to be positively regulated by c-MYC [52, 53]. In the present study, we observed that EZH2 inhibition reduced the expression of c-MYC in MM cell lines. Thus, EZH2 inhibition indirectly contributes to the downregulation of oncomiRNAs positively regulated by c-MYC such as the miR-17-92 and miR-106b-25 clusters.

Turning to the set of upregulated miRNAs that could represent direct targets of polycomb, we could show that EZH2 inhibition resulted in upregulation of miRNAs previously reported to be underexpressed in MM. This would be indicative of their potential function as tumor suppressors [46, 54]. Interestingly, in our study EZH2 inhibition also induced the expression of miRNAs that have been previously identified to be epigenetically silenced by DNA methylation in MM patients and cell lines e.g. miR-198, miR-601, miR-125a-3p and miR-320c [44, 55]. To evaluate the possibility that EZH2 inhibition upregulated miRNAs that would target the downregulated MM-associated oncogenes in this study, we used the microRNA.org resource [25]. We identified miR-125a and miR-320c as common regulators of IRF-4, XBP-1 and BLIMP-1, and to be upregulated upon EZH2 inhibition. However, this analysis did not detect any miRNAs predicted to target c-MYC to be regulated by UNC1999. We confirmed the observed upregulation of miR-125a and miR-320c in the array upon EZH2 inhibition at the primary and mature miRNA levels in two MM cell lines; INA-6 and LP-1. Furthermore, we could demonstrate that miR-125a and miR-320c are targets of EZH2 and H3K27me3 in both MM cell lines. More importantly, we could show that miR-125a and miR-320c harbor the H3K27me3 mark in MM patient cells, suggesting that they are common polycomb targets in MM.

Recently, miR-125a was shown to be underexpressed in MM as compared with normal plasma cells [54, 56]. Others have predicted miR-125a to target IRF-4 and BLIMP-1 3′UTR regions by using TargetScan and RNAhybrid software programs [57]. Gururajan et al. further demonstrated that miR-125a expression is regulated during B-cell maturation and inversely correlated with the expression of IRF-4 and BLIMP-1. The link to B-cell differentiation is interesting since miR-125a has been shown to be preferentially expressed in germinal center centroblasts but not in plasma cells [57]. This notion further supports our hypothesis that polycomb silencing of genes in MM occurs at a window of B-cell differentiation and favors growth rather than maturation as an important initial step to tumor transformation. MiR-125a-3p [55] and miR-320c [44] have been shown to be silenced by DNA methylation in MM primary cells and cell lines, and to be reactivated using DNA methylation inhibitors. Whether H3K27me3 and DNA methylation cooperate to silence miRNAs with tumor suppressor functions such as miR-125a and miR-320c in MM remains to be further elucidated, and if so, combining EZH2 and DNA methylation inhibitors could prove beneficial to target the malignant plasma cell.

In the present study, we also evaluated the effects of EZH2 inhibition on the expression of two miRNAs that were demonstrated to target c-MYC; miR-126 and miR-494. We found that EZH2 inhibition increased the expression of miR-494, but not miR-126. MiR-494 has previously been suggested to be a polycomb target and to post-transcriptionally regulate c-MYC in other B-cell tumors [58]. Recently, miRNA-494 was reported to be a part of a regulatory loop between EZH2 and c-MYC in B-cell lymphoma [59]. It is very tempting, therefore, to speculate that miRNAs operate in loops to also regulate EZH2 and other oncogenes associated to MM. One of the most interesting properties of miRNAs is their ability to target multiple molecules, frequently in the context of a network, which makes them potential therapeutic agents in tumors including MM [60, 61]. Since overexpression of a few tumor suppressor miRNAs or their mimics have shown some anti-MM activity *in vitro* [44, 55] and *in vivo* [62, 63] the potential use as anti-MM agents of miR-125a and miR-320c, identified as tumor suppressor miRNAs in this study, should be thoroughly evaluated in relevant models of MM.

In conclusion, this study supports our previous results on the potential use of EZH2 as a therapeutic target in MM. We now provide new mechanistic insights on the anti-MM effects mediated by EZH2 inhibition via upregulation of tumor suppressor miRNAs and subsequent downregulation of MM-associated oncogenes. We present for the first time a global expression profiling of miRNAs in response to EZH2 inhibition and propose EZH2 and H3K27me3 as regulators of tumor suppressor miRNAs in MM. We define miR-125a and miR-320c as polycomb targets and novel tumor suppressor miRNAs in MM, and suggest their potential therapeutic use for targeting the disease.

## MATERIALS AND METHODS

### Cell culture, treatment and reagents

All MM authenticated cell lines [64] used in this study were maintained in RPMI-1640 AQmediaTM (Sigma) supplemented with 10% FBS (Sigma), 1% GlutaMax™ (Gibco) and antibiotics (penicillin 100 U/ml and streptomycin 50 mg/ml; Sigma) at 37°C in a humidified 5% CO_2_ in-air atmosphere. The INA-6 cell line was cultured in the presence of Interleukin-6 (IL-6). For experimental setup of the 5 days viability assay, exponentially growing cells were seeded at 100 000 cells/ml overnight before addition of reagents. Medium and reagents were refreshed at day 3. UNC1999 was synthesized according to previously published procedures [65].

### Primary cells

Heparinized bone marrow samples were obtained from newly-diagnosed MM patients in accordance with the Declaration of Helsinki and approved by the local ethics committees of Uppsala and Stockholm (Dnr 2004:M-332 and 2010/1478-32). Written informed consent was obtained from each patient. Mononuclear cells were subjected to CD138 immunomagnetic purification by Whole Blood Column Kit (MACS, Miltenyi Biotec, Paris, France) according to the manufacturer's protocols. Subsequently, the purity of the CD138-enriched fraction was evaluated by May-Grünwald-Giemsa staining (Supplementary Table 3).

### Cell viability assay

MM cell lines were treated with a range of UNC1999 concentrations for 5 days with DMSO used as control treatment. On the day of analysis, cells were seeded in triplicate wells in 96-well flat-bottom plates. Cell viability was assessed using Resazurin assay reduction method using AlamarBlue (Sigma-Aldrich) as previously described [18]. Fluorescence level of at least 5 times of the blank was set as threshold for DMSO control treated sample to be included in the analysis.

### Protein extraction and Western blot

Following treatment with UNC1999 or DMSO for 5 days, MM cell lines were harvested, washed with ice-cold PBS and collected at 1500 *rpm* for 5 minutes. Total cellular protein was extracted using RIPA extraction buffer with freshly added protease inhibitor cocktail. Western blotting protocol was performed as previously described [23]. Histone proteins were extracted using the Episeeker histone extraction kit (Abcam, Ab113476) following the manufacturer's procedure. Antibodies used are listed in Supplementary Table 4.

### Gene (mRNA) expression arrays

Total RNA was extracted using TRIzol® reagent (Invitrogen) and cleaned using the RNeasy MinElute Cleanup Kit (QIAGEN). The labeling of the RNA for microarray and hybridization was done according the Affymetrix manufacturer's protocol (Affymetrix). The data was analyzed using GeneSpring 13 software and the differentially regulated genes were obtained using moderated t-test with the P-value ≤ 0.02 and fold change of 1.5.

### Mature miRNA expression arrays

Total RNA was extracted using TRIzol® reagent (Invitrogen). The microRNA arrays were performed using SurePrint G3 Human miRNA Microarray, Release 21.0, 8 × 60K format, miRBase 21.0 according to the manufacturer's protocol (Agilent Technologies). The data was analyzed using GeneSpring 13 software and the differentially regulated miRNAs were obtained using moderated t-test with the P-value ≤ 0.05 and fold change of 1.2.

### Reverse transcription and quantitative real time PCR (qRT-PCR) analysis of mRNA, primary- and mature-miRNA

For mRNA and primary-miRNA, reverse transcription using random primers (Invitrogen) was performed on 1 μg of total RNA using SuperScript^TM^ III Reverse Transcriptase (Invitrogen) according to the manufacturer's protocol. For mature-miRNA, reverse transcription was performed using TaqMan® MicroRNA Reverse Transcription Kit (Applied Biosystems) following the manufacturer's instruction. QPCR analysis of mRNA was performed using TaqMan® gene expression assays for IRF-4, XBP-1, BLIMP-1, c-MYC, EZH2 and Actin as housekeeping mRNA(Applied Biosystems). QPCR analysis of primary- and mature-miRNA was performed using TaqMan® gene expression assays for miRNA-125a, miR-320C2, miRNA-494 and RNU6B as housekeeping miRNA (Applied Biosystems).

### Chromatin immunoprecipitation (ChIP)

ChIP was performed on DMSO or UNC1999 treated cell lines and CD138^+^ purified plasma cells using a modified version of the OneDay ChIP kit (Diagenode, Liège, Belgium) as previously described [18]. Chromatin was crosslinked with 1% formaldehyde for 10 minutes at room temperature, followed by 5 minutes treatment with 1 M glycine to stop crosslinking. Chromatin was sonicated (30sec ON/30 sec OFF) for 6 cycles of 5 minutes each at ultrasonic wave output power 320 W in Bioruptor® (Diagenode, Liège, Belgium). Cells were collected and lysed on ice in RIPA extraction buffer with a cocktail of protease inhibitors. Antibodies used are listed in Supplementary Table 4. Precipitated DNA was analyzed by Real Time-qPCR using Platinum® SYBR® Green qPCR SuperMix UDG with Rox (Invitrogen, Carlsbad, CA) and 0.25 mM of each forward and reverse primers. Primer sequences used in this study were, miR-125a: forward primer: 5´-CCCTTCCTCCAGAGCATGAC-3` and reverse primer: 5´-CCATCGTGTGGGTCTCAAGG-3´; miR-320c2: forward primer: 5´-GTTGAGGAGCACTGGGTATGT-3´and reverse primer: 5´-CTTACCCTCTCAACCCA GCTT-3´. The PCR conditions were: 95°C for 2 min followed by 40 cycles of 95°C for 0:30 min and 60°C for 1 min. The run and analysis were performed using Mx3005P instrument and software (Stratagene).

### Statistical analysis

Paired, two-tailed Student t-test was calculated using GraphPad Prism, *: P-value≤0.05, **: P-value≤0.01, ***: P-value≤0.001.

### Accession numbers

The accession number for the raw and processed array data reported in this paper is GEO: GSE87716, with the subseries accession numbers GEO: GSE87714 and GEO: GSE87715 for mRNA arrays and miRNA arrays, respectively.

## SUPPLEMENTARY MATERIALS FIGURES AND TABLES






