# Supplementary material for: EZH2 inhibition in multiple myeloma downregulates myeloma associated oncogenes and upregulates microRNAs with potential tumor suppressor functions
Source: Oncotarget. 2016 Dec 30;8(6):10213–24. doi: 10.18632/oncotarget.14378 (PMC5354653; doi:10.18632/oncotarget.14378)
Supplement: Supplementary file 1 [file oncotarget-08-10213-s001.pdf]

EZH2 inhibition in multiple myeloma downregulates myeloma associated oncogenes and upregulates microRNAs with potential tumor suppressor functions

SUPPLEMENTARY DATA

SUPPLEMENTARY MATERIALS AND METHODS

Caspase activation assay

Cells were seeded at a density of 100 000 cells/ml overnight before addition of reagents. INA-6 and KMS-11 MM cell lines were treated with 1µM or 4µM UNC1999, respectively, or with DMSO as control for 5 days. Medium and reagents were refreshed at day 3. Caspase-3 and -7 activity was measured every 24h using the CellEvent™ caspase-3/7 green detection reagent (Life Technologies) following the manufacturer's protocol.

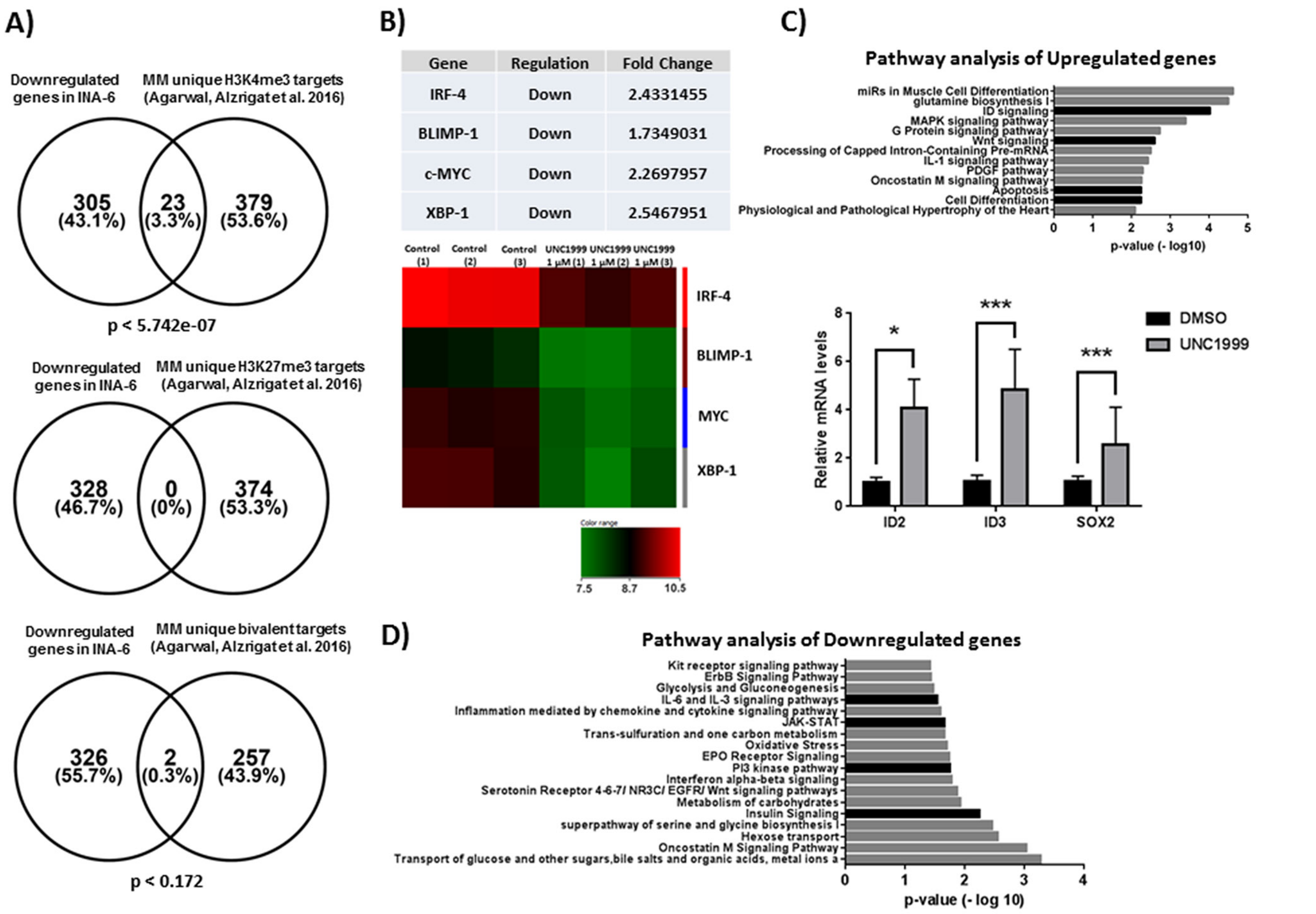

Supplementary Figure 1: UNC1999 downregulated genes are enriched among MM H3K4me3 targets. A. UNC1999 downregulated genes in the MM INA-6 cell line are enriched among previously defined H3K4me3 targets unique to MM, as compared with normal plasma cells, but not among MM-unique H3K27me3 and bivalent targets in MM. **B.** UNC1999 mediated inhibition of EZH2 downregulates the expression of MM-associated oncogenes; IRF-4, BLIMP-1, c-MYC and XBP-1. **C.** Pathway analysis of upregulated genes and qPCR validation of some selected genes involved in pathways such as apoptosis i.e. ID2 and ID3 and cell differentiation i.e. SOX2. Actin was used as housekeeping gene. **D.** Pathway analysis of downregulated genes upon EZH2 inhibition in the INA-6 cell line. For all experiments, the MM INA-6 cell line was treated with 1µM of UNC1999 for 5 days. DMSO was used as control treatment. P-values were calculated using student t-test GraphPad prism, P-value: *≤ 0.05; ***≤0.001.

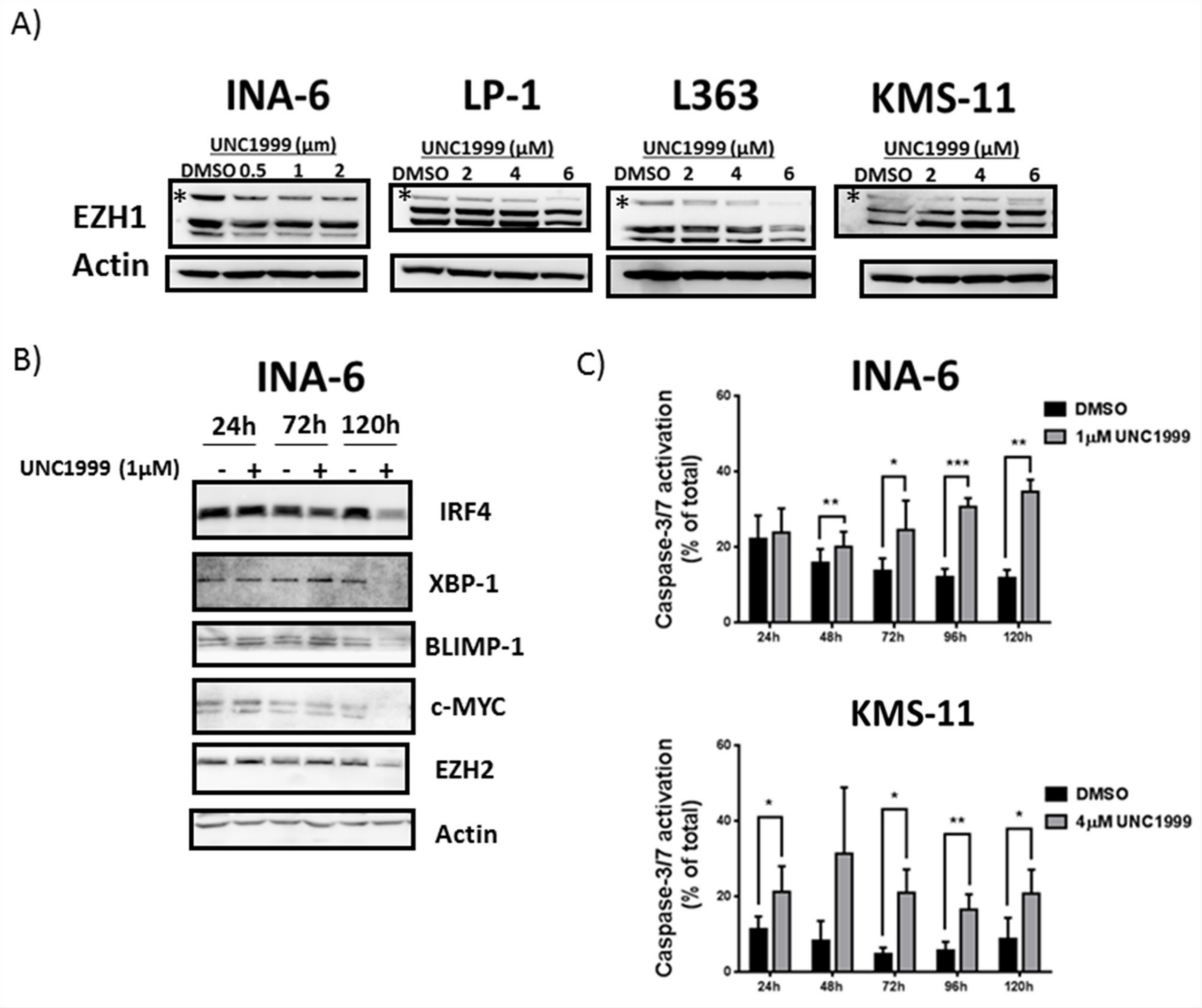

Supplementary Figure 2: Long-term treatment with UNC1999 is required to downregulate MM associated oncogenes. **A.** Long-term treatment (5 days) of INA-6 cell line with 1µM of UNC1999 downregulated EZH1 protein levels. MM cells were treated with a range of UNC1999 concentrations or DMSO for 5 days. * indicates the 95 KDa band of EZH1. **B.** Kinetics of protein expression over time upon treatment of UNC1999 in the MM INA-6 cell line indicated that long-term treatment with UNC1999 (5 days) is required to downregulate MM-associated oncoproteins. INA-6 was treated with 1µM for the indicated time points. DMSO was used as control treatment. Western blots are representative of three independent biological experiments. **C.** UNC1999 induces a time dependent increase in caspase-3 and -7 activity in INA-6 and KMS-11 cell lines. INA-6 and KMS-11 were treated with 1 µM or 4 µM UNC1999, respectively, and percentage of cells with activated caspase-3/7 was measured using the CellEvent™ caspase-3/7 green detection reagent (Life Technologies). DMSO was used as control treatment. Error bars represent standard deviation of four independent biological experiments. P-values were calculated using student t-test GraphPad prism, P-value: *≤ 0.05; **≤0.01; ***≤0.001.

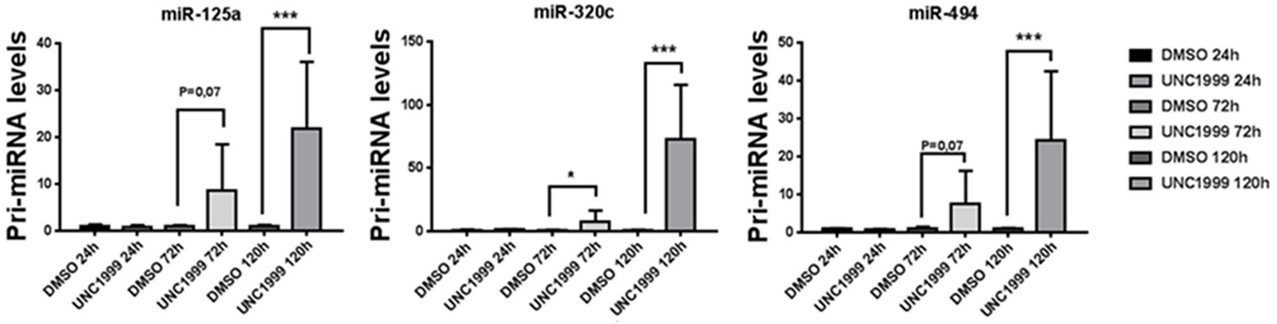

Supplementary Figure 3: Long-term inhibition of EZH2 is required for reactivation of microRNA genes. UNC1999 reactivated the expression of microRNA genes as early as 72 hours post-treatment and led to a significant reactivation upon long-term treatment for 5 days. QPCR analysis of primary microRNA in INA-6 cell line treated with 1 μM for the indicated time points. DMSO was used as control treatment. RNU6B was used as a house keeping control. Error bars represent standard deviation of three independent biological experiments. P-values were calculated using student t-test GraphPad prism, P-value: *≤ 0.05; ***≤0.001.

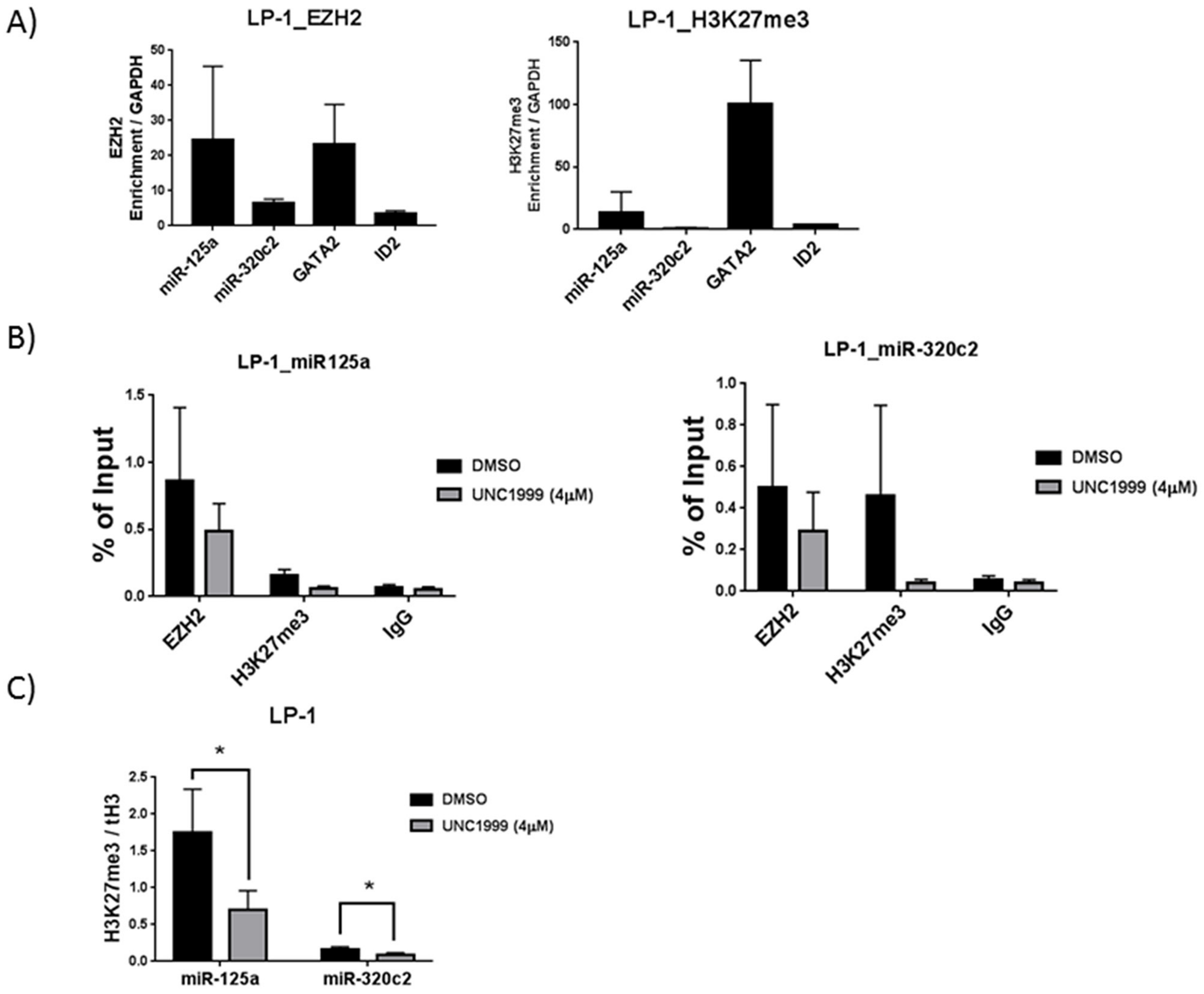

Supplementary Figure 4: MiR-125a and miR-320c are polycomb targets in MM cells. EZH2 and H3K27me3 occupancy was investigated at the miR-125a and miR-320c gene bodies in the LP-1 cell line. **A.** EZH2 and H3K27me3 enrichment at miRNAs gene bodies. GATA2 and ID2 promoter regions were used as positive control for enrichments, while the GAPDH promoter region was used as negative control. Enrichment was represented relative to the negative control, GAPDH. **B.** EZH2 inhibition using UNC1999 reduced the occupancy of EZH2 and H3K27me3 mark at miR-125a and miR-320c gene bodies. **C.** Reduced levels of H3K27me3 mark at miR-125a and miR-320c upon UNC1999 treatment was independent of total histone H3 levels. LP-1 cell line was treated with 4 µM of UNC1999 for 5 days. DMSO was used as control treatment. Error bars represent standard deviation of three independent biological experiments. P-values were calculated using student t-test GraphPad prism, P-value: *≤ 0.05.

Supplementary Table 1: UNC1999 differentially regulated mRNAs

See Supplementary File 1

Supplementary Table 2: UNC1999 differentially regulated miRNAs

See Supplementary File 2

Supplementary Table 3: Disease status of patients from which CD138+ malignant plasma cells were used for ChIP analysis

Patient	Disease stage	Ig Isotype	% of plasma cells
1	MGUS	IgG/□	59
2	Multiple Myeloma / ISS stage II	IgG/□	90
3	Multiple Myeloma / ISS stage III	IgG/□	94
4	Multiple Myeloma / ISS stage II	IgG/□	98
5	Multiple Myeloma / ISS stage I	IgG/□	100
6	Multiple Myeloma / ISS stage II	IgG/□	98

Supplementary Table 4: Antibodies used in this study

Antibody	Application	Provider
IRF-4	Western Blot (1:500 dilution)	Santa Cruz (M-17): sc-6059
XBP-1	Western Blot (1:500 dilution)	Santa Cruz (M-186): sc-7160
BLIMP-1	Western Blot (1:500 dilution)	Santa Cruz (C-7): sc-398699
EZH2	Western Blot (1:1000 dilution)	Cell Signaling (AC22)
c-MYC	Western Blot (1:1000 dilution)	Abcam (Ab11917)
Actin	Western blot (1:1000 dilution)	Santa Cruz (I-19): sc-1616
EZH2	ChIP	Millipore (AC22): Cat.#17-662
EZH1	Western Blot (1:3000 dilution)	Millipore (ABE281)
H3K27me3	ChIP and Western Blot (1:1000 dilution)	Millipore Cat.#07-449
H3	ChIP and Western Blot (1:1000 dilution)	Abcam (Ab1791)
IgG control	ChIP	Millipore (AC22): Cat.#17-662
